# Association of circulating leptin and adiponectin with periodontitis: a systematic review and meta-analysis

**DOI:** 10.1186/s12903-017-0395-0

**Published:** 2017-06-29

**Authors:** Junfei Zhu, Bin Guo, Xueqi Gan, Ling Zhang, Yuting He, Beilei Liu, Xin Chen, Suhan Zhang, Haiyang Yu

**Affiliations:** 1 State Key Laboratory of Oral Diseases, Sichuan University, Chengdu, 610041 China; 2 Institute of Stomatology of Chinese PLA General Hospital, 28 Fuxing Road,Haidian District, Beijing, 100853 People’s Republic of China; 3 West China Medical School, Sichuan University, Chengdu, 610041 China; 4 Department of Prosthodontics, West China Hospital of Stomatology, Sichuan University, Chengdu, 610041 China

**Keywords:** Periodontitis, Periodontal treatment, Leptin, Adiponectin, Meta-analysis

## Abstract

**Background:**

This study aimed to assess the difference in serum levels of leptin and adiponectin in patients with periodontitis and in periodontally healthy individuals and evaluate the changes in circulating leptin and adiponectin after periodontal therapy. Leptin and adiponectin are the most generally studied adipokines that function as inflammatory cytokines. Although the association between periodontitis and serum levels of leptin and adiponectin has been studied extensively, the results were not consistent.

**Methods:**

A systematic search of the Pubmed, Embase, Web of Science, and Cochrane Library up to September 2016 was conducted. The studies were screened and selected by two writers according to the specific eligibility criteria. The quality of included cross-sectional studies was assessed using the quality assessment form recommended by the Agency for Healthcare Research and Quality and Methodological Index for Nonrandomized Studies. The meta-analyses were conducted using the STATA 12.0 software.

**Results:**

A total of 399 manuscripts were yielded and 25 studies were included in the present meta-analysis. Significantly elevated serum levels of leptin and decreased serum levels of adiponectin in patients with periodontitis were observed in the subgroup analysis of body mass index (BMI) <30. The overall and subgroup analyses showed no significant change in the serum levels of leptin in patients with periodontitis after periodontal treatment. The subgroup analysis of systemically healthy patients showed no significant change in serum levels of adiponectin in patients with periodontitis after periodontal treatment.

**Conclusions:**

The present meta-analysis supported elevated serum levels of leptin and decreased serum levels of adiponectin in patients with periodontitis compared with controls in the BMI <30 population. In systemically healthy patients with periodontitis, serum levels of leptin and adiponectin do not significantly change after periodontal treatment.

## Background

Periodontitis is a common progressive inflammatory disease affecting human periodontal support tissues [1]. The development of periodontitis cause a series of clinical manifestations including gingival bleeding, periodontal pocket formation, alveolar bone absorption, and eventually teeth loss [2]. The progression of chronic periodontitis (CP) is mediated by both bacterial invasions and host immunoinflammatory responses [3–5]. The immunocytes activated by the exogenous bacteria not only destroy bacteria but also release myriads of products, known as inflammatory cytokines, which exert an anti-bacterial effect and, on the contrary, contribute to the destruction of periodontal tissues [6, 7]. Hence, considerable attention was paid in drawing an association between periodontitis and certain inflammatory cytokines, such as interleukin-1 (IL-1), tumor necrosis factor-α (TNF-α), and matrix metalloproteinase-8 (MMP-8). Moreover, the presence of such inflammatory cytokines has been proved to be altered in serum, gingival crevicular fluid, and saliva in patients with periodontitis [8–11].

Adipokines are a group of bioactive molecules primarily secreted by adipose tissues [12]. Adipokines, such as leptin, adiponectin, resistin, and visfatin, are important in periodontal inflammation for functioning as proinflammatory and anti-inflammatory cytokines [13]. Of all these adipokines, leptin and adiponectin have been extensively described because of their critical role in immune response, bone and lipid metabolism, energy expenditure, and insulin sensitivity modulation [14]. Leptin, a 16-kDa nonglycosylated peptide hormone expressed by the obese gene, has been considered to be the most generally studied adipokine [15, 16]. Besides being known as an appetite controller, leptin exerts roles in both systemic and local inflammatory processes [17–20]. Leptin is considered to be a proinflammatory cytokine involved in the inflammatory response as it modulates the function of immunocytes such as T-cells, monocytes, and natural killer cells. These immunocytes are directly activated by leptin, leading to an elevation in the release of other inflammatory mediators [19, 21]. Inversely, adiponectin, the insulin-sensitizing adipokine, mediates anti-inflammatory effects in the process of inflammation and functions in cell proliferation, differentiation, and regeneration [13, 22]. Recent studies have reported that adipokines, such as leptin and adiponectin, are produced in periodontal cells and contribute to periodontal infection and healing [23, 24]. Additionally, adipokines are also revealed to play roles in periodontitis-related systemic conditions such as diabetes and obesity [25–27]. In this light, to clarify the association between adipokines and periodontitis can be conducive to investigate not only the mechanism of periodontal inflammations but also the impact of periodontitis on systemic diseases.

Although multiple studies have been conducted to find the association between periodontitis and serum levels of leptin and adiponectin, the findings were inconsistent [16, 28–32]. Moreover, no meta-analyses have been reported on this issue so far. Therefore, the present systematic review and meta-analysis were performed to summarize individual study results into a quantitative estimation of the association between periodontitis and serum levels of leptin and adiponectin. An evidence-based result would be valuable in providing a more precise evaluation of the association between adipokines and periodontitis. The clinically focused questions of present study are: (1) Will serum levels of leptin and adiponectin be significantly altered in patients with periodontitis comparing with periodontally healthy individuals? (2) Do serum levels of leptin and adiponectin change significantly after periodontal treatment?

## Methods

The review protocol was prospectively registered at the National Institute for Health Research PROSPERO, International Prospective Register of Systematic Reviews under registration CRD42016047213. The present study was conducted in accordance with the Preferred Reporting Items of Systematic Reviews and Meta-Analyses statement [33].

### Eligibility criteria

The inclusion criteria for the studies comparing the difference in serum levels of leptin and adiponectin between patients with periodontitis and periodontally healthy individuals were as follows: (1) the studies were case-controlled, cross-sectional, prospective, or clinical trials; (2) the periodontitis groups consisted of patients diagnosed with periodontal disease, including chronic periodontitis (CP) and aggressive periodontitis (AP). Present study employed the definition of periodontitis as: at least 1 sites with probing pocket depth (PPD) ≥4 mm [34]; (3) the healthy groups consisted of periodontally healthy individuals; and (4) serum levels of leptin and adiponectin were detected and provided in both the periodontitis and healthy groups.

The inclusion criteria for the studies evaluating the change in circulating leptin and adiponectin after periodontal treatment were as follows: (1) the studies were randomized controlled trials, nonrandomized controlled trials, or prospective; (2) patients with periodontitis were enrolled; (3) periodontal treatments such as scaling and root planning and periodontal surgeries with or without antibiotics administration were applied; and (4) serum levels of leptin and adiponectin of the patients with periodontitis at baseline and after the periodontal treatments were provided.

The exclusion criteria were as follows: (1) the studies were review articles, case reports, letters, or conference abstracts; (2) studies with insufficient data for the statistical analyses; and (3) those with repeated data.

### Search strategy and record screen

Pubmed, Embase, Web of Science, and Cochrane Library up to September 2016 were systemically searched to identify the pertinent studies. The search strategies are presented in Table 1. In addition, the reference lists of the selected manuscripts and related reviews were also manually searched and screened for a comprehensive search result.

**Table 1:** Search strategy

Pubmed:	Embase:	Web of science:	Cochrane library:
((((“Periodontal Diseases”[Mesh] OR “Periodontitis”[Mesh]) OR “Dental Scaling”[Mesh]) OR “Periodontics”[Mesh]) OR (((((((periodontal disease[Title/Abstract] OR periodontitis[Title/Abstract]) OR periodontal pocket[Title/Abstract]) OR periodontal tissue[Title/Abstract]) OR periodontal therapy[Title/Abstract]) OR periodontal treatment[Title/Abstract]) OR scaling and root planning[Title/Abstract]) OR SRP[Title/Abstract]) AND ((((leptin[Title/Abstract] OR adiponectin[Title/Abstract]) OR adipokine[Title/Abstract]) OR adipocytokine[Title/Abstract]) OR ((“Adipokines”[Mesh] OR “Leptin”[Mesh]) OR “Adiponectin”[Mesh]))	‘periodontal disease’/exp OR ‘periodontitis’/exp OR ‘periodontics’/exp OR ‘periodontal disease’:ab,ti OR ‘periodontitis’:ab,ti OR ‘periodontal pocket’:ab,ti OR ‘periodontal tissue’:ab,ti OR ‘periodontal therapy’:ab,ti OR ‘periodontal treatment’:ab,ti OR ‘scaling and root planning’:ab,ti OR ‘srp’:ab,ti AND (‘leptin’/exp OR ‘adiponectin’/exp OR ‘adipocytokine’/exp OR ‘leptin’:ab,ti OR ‘adiponectin’:ab,ti OR ‘adipokine’:ab,ti OR ‘adipocytokine’:ab,ti)	#1:TS = (periodontal disease) OR TS = (periodontitis) OR TS = (periodontal treatment) OR TS = (periodontal therapy) OR TS = (SRP) OR TS = (scaling and root planning) OR TS = (periodontal pocket) OR TS = (periodontal tissue) #2:TS = (adipokine) OR TS = (adipocytokine) OR TS = (leptin)OR TS = (adiponectin) #1 AND #2	#1: “periodontal disease”:ti,ab,kw; #2:“periodontitis”:ti,ab,kw; #3: periodontal treatment:ti,ab,kw; #4: periodontal therapy:ti,ab,kw; #5: scaling and root planning:ti,ab,kw; #6: SRP:ti,ab,kw; #7: “periodontal pocket”:ti,ab,kw; #8: periodontal tissue:ti,ab,kw; #9: #1 or #2 or #3 or #4 or #5 or #6 or #7 or #8; #10:“adipokine”:ti,ab,kw; #11: “adipocytokine”:ti,ab,kw; #12: “leptin”:ti,ab,kw; #13:“adiponectin”:ti,ab,kw; #14: #10 or #11 or #12 or #13; #15 :#9 and #14

According to the eligibility criteria, titles and abstracts were screened first and the full-text paper screen was conducted next. The results were screened by two authors (Xin Chen and Ling Zhang) independently, and a third author (Yuting He) was consulted if any discrepancy existed.

### Data extraction

The following data were extracted from the included studies by two authors (Junfei Zhu and Ling Zhang) independently, and discrepancies were resolved through discussion: (1) Name of the first author and year of publication; (2) study design; (3) country and ethnicity; (4) group size; (5) body mass index (BMI); (6) age; (7) outcomes; (8) systemic conditions; (9) smoking status; (10) posttherapy time; and (11) serum levels of leptin and/or adiponectin.

### Quality assessment

The methodological quality of the included studies was evaluated by two authors (JF Zhu and L Zhang) independently, and discrepancies were resolved through discussion. The quality of the included cross-sectional studies was assessed using the quality assessment form for cross-sectional studies recommended by the Agency for Healthcare Research and Quality (AHRQ). A total of 11 items were involved in this form, and 1 point could be achieved if the item was reflected in the study. No points could be achieved if the item was not considered or unclear. The final scores ranged from 0 to 11. Studies with scores 0–4, 5–8, and 9–11 were considered as of low, moderate, and high quality, respectively [35, 36]. In addition, for the studies evaluated the changes in serum levels of leptin and adiponectin after periodontal treatment, the included double-arm clinical trials and randomized controlled clinical trials were treated as single-arm clinical trial designs. Therefore, the Methodological Index for Nonrandomized Studies (MINORS) was applied for assessing the quality of the included clinical trials [37]. A total of 12 items were involved in MINORS, and 0–2 points could be achieved in each of the items. The first eight items were designed for the studies without a control group,and the other four items are the additional criteria for comparative studies. As in present quality assessment we only evaluated the quality of single-arm designs, the first 8 items were employed. The final scores ranged from 0 to 16. Studies with scores 0–5, 6–10, and 11–16 were considered as of low, moderate, and high quality, respectively [38].

### Data Analyses

Four models were considered in the present meta-analysis: serum levels of leptin in patients with periodontitis versus healthy individuals (L1), serum levels of adiponectin in patients with periodontitis versus healthy individuals (A1), serum levels of leptin in patients with periodontitis before versus after periodontal treatment (L2), and serum levels of adiponectin in patients with periodontitis before versus after periodontal treatment (A2). The data on serum levels of leptin and adiponectin were presented as mean (M) ± standard deviation (SD). If the outcomes were provided only as median (minimum – maximum) [39–41], the results were shifted to the form of M ± SD according to the estimation method reported by Hozo et al. [42]. The standard mean difference (SMD) and corresponding 95% confidence interval (CI) were applied to estimate the association between serum levels of leptin and adiponectin and periodontitis. Heterogeneity was estimated by chi-square and *I*; a *P* value <0.05 and *I* ^2^ > 50% were considered significant heterogeneity [43], and then the random-effects model was used; otherwise, a fixed-effects model was used to assure statistical efficiency. Subgroup analyses were conducted according to the type of periodontitis, BMI, smoking status, systemic conditions, and types of periodontal treatment. The following characteristics were included as covariates in the meta-regression to explore the potential sources of heterogeneity: type of periodontitis, BMI, smoking status, and presence of systemic disease. A characteristic was considered as the source of heterogeneity if *P* <0.05. Moreover, Galbraith plots were conducted to investigate the heterogeneity. Sensitivity analyses were performed to test the robustness of the results. Publication bias was measured by Begg’s and Egger’s linear regression tests. A significant publication bias was considered if the *P* value was <0.05. All statistical analyses were processed using the STATA 12.0 software.

## Results

### Study selection

**Fig. 1:**
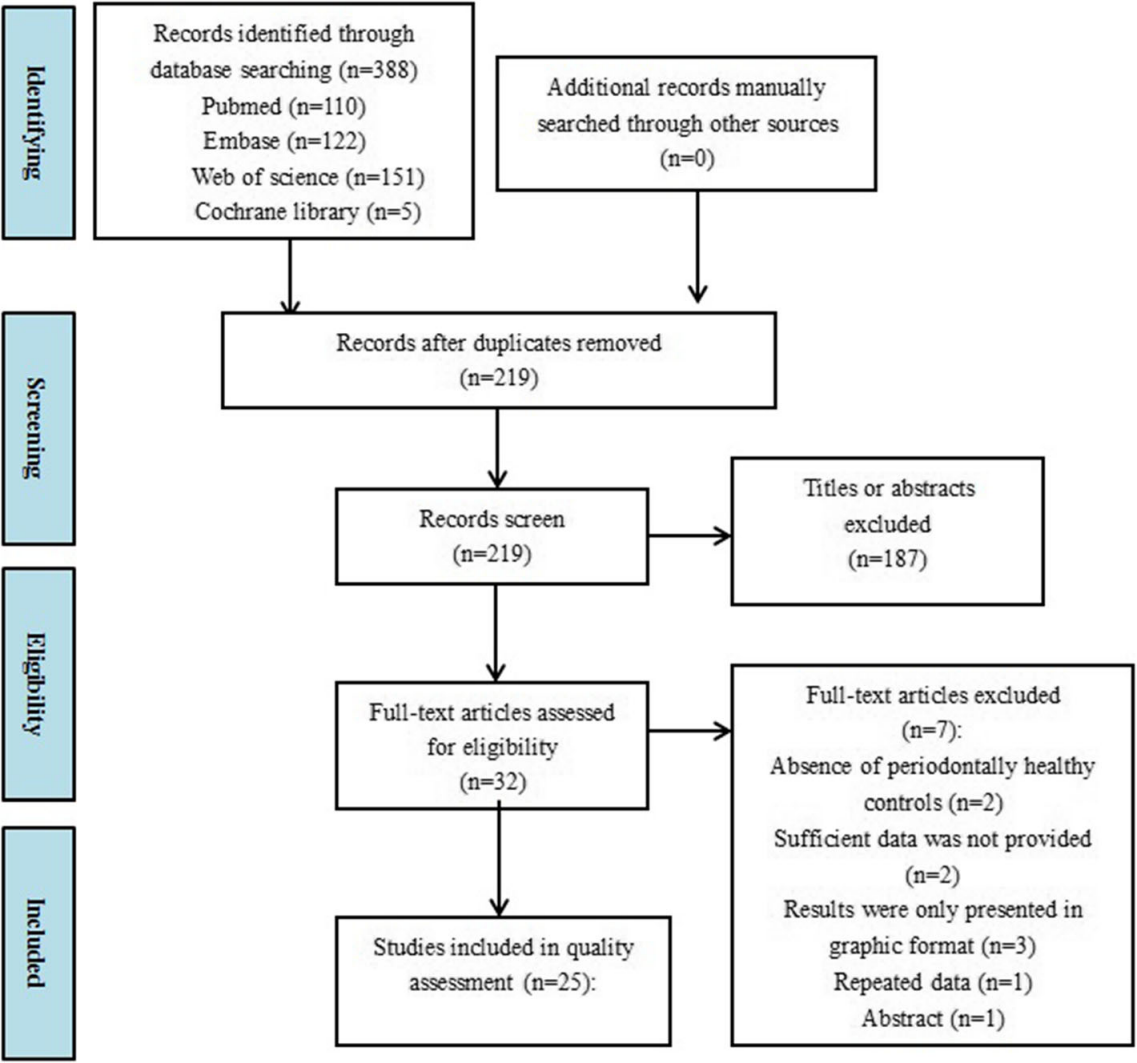
Study selection

A total of 399 manuscripts were yielded using the search strategy. After deleting the duplicates, full-text screens were conducted and 25 studies were identified to be eligible. The process of study selection and the reasons for exclusion are listed in Fig. 1.

### Characteristics and quality assessment

Of the 25 included studies, all 16 studies focusing on the difference in serum levels of leptin and/or adiponectin between patients with periodontitis and healthy individuals employed the cross-sectional design [16, 30–32, 40, 41, 44–53]. Besides this, nine clinical trials detecting the serum levels of leptin and adiponectin in patients with periodontitis before and after periodontal treatments were included [28, 29, 39, 54–59]. The clinical trials performed by Duzagac et al., Purwar et al., and Shimada et al. also enrolled the periodontally healthy groups and provided the pretherapy data on the serum levels of leptin and adiponectin in patients with periodontitis and healthy individuals [54, 57, 58]. The studies performed by Altayet al., Duzagacet al., Goncalveset al, Mendoza-Azpuret al, and Zimmermann et al. provided the data on subjects with BMI <30 and BMI ≥30 separately [31, 32, 39, 54–56], and the study performed by Zeigler et al. provided only the data on obesity subjects. The study performed by Ay et al. enrolled both the CP and aggressive periodontitis (AP) groups [40]. The study performed by Gundala et al. enrolled both the systemically healthy group and the acute myocardial infarction (AMI) group [47]. The study performed by Davies et al. provided the data on male and female subjects separately [44]. For the included cross-sectional studies, the AHRQ scores ranged from 5 to 7. The study performed by Gangadhar et al. was the only cross-sectional study considered to be of low quality (4 points) [46]. Other included cross-sectional studies were all considered to be of moderate quality. All of the included clinical trials were considered to be of high quality. The characteristics and quality assessment of the included studies are presented in Table 2.

**Table 2:** Study charicteristics

Authors and years	Study design	Country (Ethnicity)	Type of periodontitis	Group size (PG/HG)	Age(PG/HG)	BMI(PG/HG)	Systemic disease	Smoking	Adipokine	Quality assessment	
Ay, Z. Y. 2012	Cross-sectional	Turkey (Caucasion)	CP/AP	21/20	47/37/33	28/27.6/24.39	Free	Free	Leptin	●●●○●●●○○○○(6/12)	
Ay, Z. Y 2013	Cross-sectional	Turkey (Caucasion)	AP	6/6	38.67/32.33	25.78/23.20	Free	Free	Leptin	●●●○●●●●○○○(7/12)	
Cecilia C Z 2015	Cross-sectional	Sweden (Caucasion)	P	14/61	14.5/14.5	35.9/37.4	Not mentioned	Mixed	Leptin/Adiponectin	●●●○●●●●○○○(7/12)	
Davies, R. C. 2011	Cross-sectional	UK (Caucasion)	AP	30/30	36.7/36.3	26.0/25.3	Free	Mixed	Leptin/Adiponectin	●●○○●●●●○○○(6/12)	
Furugen, R 2008	Cross-sectional	Japan(Asian)	CP	84/70	76/76	22.77/22.39	Not mentioned	Mixed	Adiponectin	●●●○●●●●○○○(7/12)	
Gangadhar, V.2011	Cross-sectional	India(Indian)	P	25/25	18-60	Not mentioned	Not mentioned	Mixed	Leptin	●●○○●○●○○○○(4/12)	
Gundala, R.2014	Cross-sectional	India(Indian)	CP	30/30;30/30	44.9/48.3;48.9/47.8	27.4/21.7;30.8/21.6	Free/AMI	Mixed	Leptin	●●●○●●●○○○○(6/12)	
Karthikeyan, B. V 2007	Cross-sectional	India(Indian)	CP	14/14	30–39 matiched	Normal BMI	Not mentioned	Mixed	Leptin	●●○●●●●●○○○(7/12)	
Mendoza-Azpur 2015	Cross-sectional	Peru (Peruvian)	CP	19/29;24/21	41.4/42.5;47.2/26.9	Normal BMI; Obesity	Free	Free	Leptin/Adiponectin	●●●○●●●●○○○(7/12)	
Nagano, Y 2011	Cross-sectional	Japan(Asian)	CP	62/89	55.7/50.7	24.4/24.2	Not mentioned	Mixed	Adiponectin	●●○●●○●●○○○(6/12)	
Purwar, P.A 2015	Cross-sectional	India(Indian)	CP	44/40	49.3/49.9	20.62/20.69	Free	Free	Leptin	●●●○●●●●○○○(7/12)	
Saito, T 2008	Cross-sectional	Japan(Asian)	CP	34/42	55.2/55.1	23.3/21.9	Not mentioned	Mixed	Adiponectin	●●●●●●●○○○○(7/12)	
Sete, M. R.	Cross-sectional	Brazil (Brazilian)	CP	15/15	45.7/32.1	28.8/Not mentioned	Free	Free	Leptin/Adiponectin	●●○○●●●○○○○(5/12)	
Shi, D 2015	Cross-sectional	China (Asian)	AP	90/44	26.2/25.6	21.9/21.1	Free	Mixed	Leptin	●●●○●●●●○○○(7/12)	
Thanakun, S.2014	Cross-sectional	Thai (Asian)	CP	58/67	50.0/46.0	24.9/23.5	Mixed	Mixed	Leptin/Adiponectin	●●●○●●●●○○○(7/12)	
Zimmermann, G. S2013	Cross-sectional	Brazil (Brazilian)	CP	20/20;20/18	47.8/42.9;51.2/43.2	23.0/23.4;33.2/33.9	Free	Free	Leptin/Adiponectin	●●●○●●●●○○○(7/12)	
Authors and years	Study design	Country (Ethnicity)	Group size	Age(NG/OBG)	BMI(NG/OBG	Systemic disease	Smoking	Adipokine	Periodontal treatment	Quality assessment	Time interval
Altay, U. A 2013	Double-arm clinical trial	Turkey (Caucasion)	24/22	42.5/45.6	26.3/32.2	Free	Mixed	Leptin	SRP	●◐●●◐●◐●(13/16)	3 month
Behle, Jan H 2009	Single-arm clinical trial	USA (mixed)	28	43.3	Not mentioned	Free	Free	Adiponectin	SRP + Periodontal surgery	●◐●●◐◐●◐ (12/16)	1 month
Bharti, P 2013	Controlled clinical trial	Japan(Asian)	21	60.2	24.2	T2DM	Free	Adiponectin	SRP + Topical antibiotics	●◐●●◐●◐◐(12/16)	2 month
Duzagac, E 2015	Double-arm clinical trial	Turkey (Caucasion)	15/15	41.06/40.66	22.14/36.26	Free	Free	Adiponectin	SRP	●◐●●◐●●◐ (13/16)	3 month
Goncalves, T. E A 2015	Double-arm clinical trial	Brazil (mixed)	24/24	48.4/48.8	24.4/33.2	Free	Free	Leptin/Adiponectin	SRP	●●●●◐●◐●(14/16)	3 month
Goncalves, T. E B 2015	Double-arm clinical trial	Brazil (mixed)	20/20	48.5/50.0	23.4/36.1	Free	Free	Leptin/Adiponectin	SRP	●●●●◐●●●(15/16)	3 month
Purwar, P.B 2015	Single-arm clinical trial	India(Indian)	22	35-60	20.45	Free	Free	Leptin	SRP	●◐●●◐●◐●(13/16)	3 month
Shimada, Y.2010	Single-arm clinical trial	Japan(Asian)	33	55.1	22.5	Free	Mixed	Leptin	SRP	●◐●●◐◐●◐(12/16)	1 month
Sun, W. L2011	Randomized controlled clinical trial	China(Asian)	82	55.13	23.71	T2DM	Free	Adiponectin	SRP + Periodontal surgery	●◐●●◐●●◐ (13/16)	3 month

*PG* Periodontitis group, *HG* Healthy group, *NG* Non-obese group, *OG* Obese group. *AP* Aggressive periodontitis, *CP* chronic periodontitis, *P* periodontitis, *T2DM* type 2 diabetes mellitus, *SRP* scaling and root planning; The details of quality assessment were described with stars. Each star stands for one item in the assessment form (AHRQ or MINORS) in order. ● = the item was reported and adequate; ○ = the item was not reported; ◐ = the item was reported but inadequate

### Overall and subgroup analyses

**Fig. 2:**
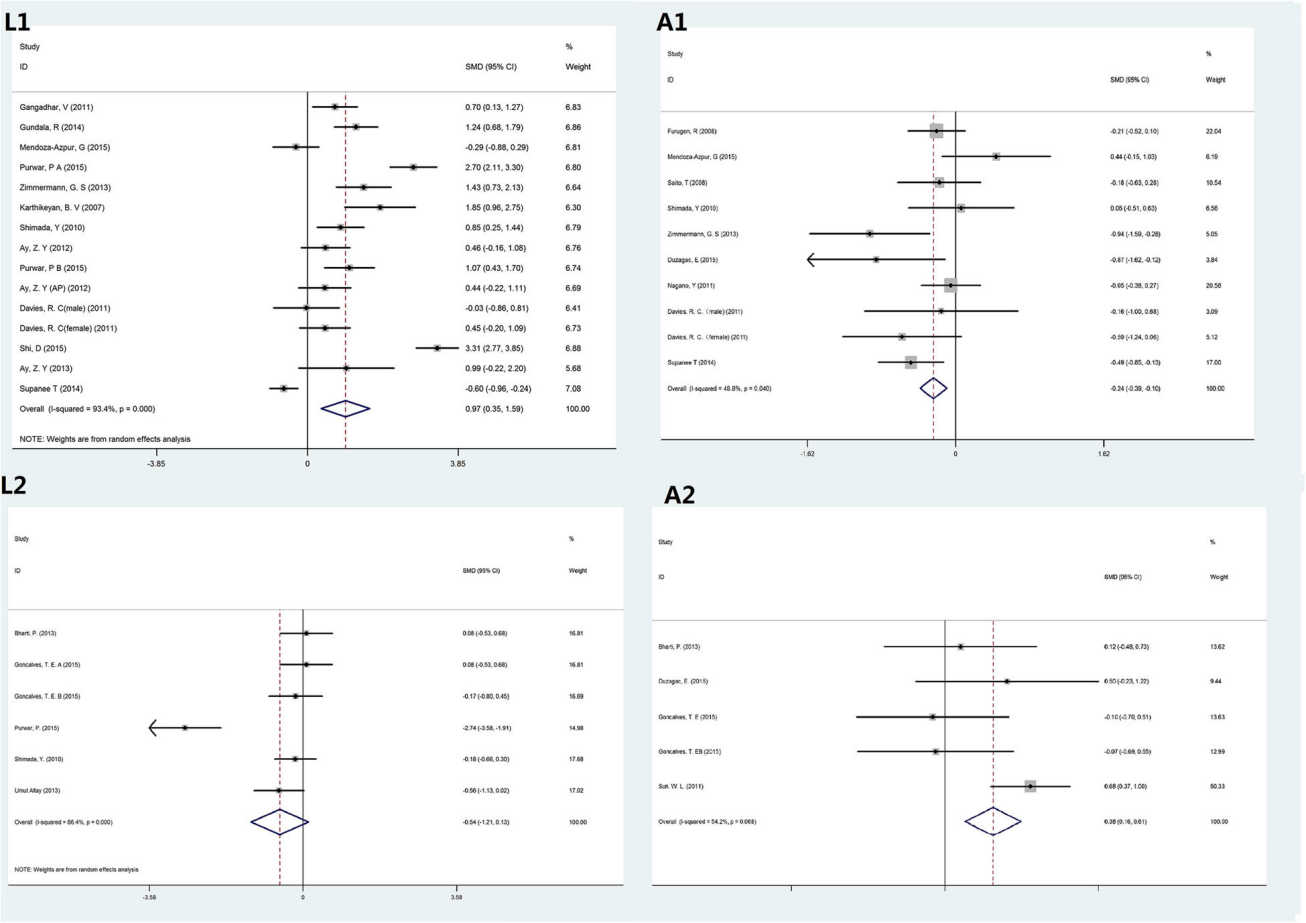
Forest plot of standard mean difference (SMD) with 95% confidence intervals (CIs) of the L1, A1, L2, A2 models in the subgroup of BMI <30

In the L1 model, higher serum levels of leptin were observed in the overall and subgroup analyses of BMI <30. The subgroup analysis of BMI ≥30 did not show significant results. The overall and subgroup analyses of the L1 model demonstrated significant heterogeneity. In the A1 model, the overall and subgroup analyses showed a significantly decreased serum level of adiponectin in patients with periodontitis, except for the subgroup of BMI ≥30. In the L2 model, the overall and subgroup analyses showed no significant change in serum levels of leptin in patients with periodontitis after periodontal treatment. In the A2 model, the overall and subgroup analyses of BMI <30 showed a significantly elevated level of serum adiponectin in patients with periodontitis after periodontal treatment. However, no significant result was obtained by the subgroup analysis of BMI ≥30 and the subgroup analyses based on the methods of periodontal treatment and systemic conditions. The results of overall and subgroup analyses are summarized in Table 3, and the forest plots of the subgroup analyses of BMI <30 are presented in Fig. 2.

**Table 3:** The overall and subgroup analyses

Overall and subgroup analyses	No. of observations	Association		Heterogeneity		Model	Publication bias (*P*)
		SMD (95% CI)	*p* value	*I* ^2^ (%)	*P* value		
L1	20	0.799 (0.308–1.289)	0.000	92.2	0.000	Random	0.289
BMI <30	14	0.987 (0.317–1.657)	0.000	93.9	0.000	Random	
BMI ≥30	3	0.024 (−0.790–0.839)	0.953	81.3	0.005	Random	
CP	13	0.708 (0.142–1.273)	0.014	91.6	0.000	Random	
AP	5	1.046 (−0.366–2.458)	0.147	94.8	0.000	Random	
Systemic disease (free)	15	0.822 (0.225–1.418)	0.007	92.1	0.000	Random	
Systemic disease (mixed)	5	0.721 (−0.135–1.576)	0.099	91.6	0.000	Random	
Smoking (free)	9	0.656 (−0.076–1.389)	0.079	90.6	0.000	Random	
Smoking (mixed)	11	0.913 (0.223–1.602)	0.009	93.6	0.000	Random	
A1	14	−0.323 (−0.525 to −0.121)	0.002	50.2	0.016	Random	0.203
BMI <30	10	−0.243(−0.390 to −0.096)	0.001	48.8	0.040	Fixed	
BMI ≥30	3	−0.326 (−0.677–0.024)	0.068	33.9	0.220	Fixed	
CP	12	−0.317 (−0.540 to −0.095)	0.005	56.3	0.009	Random	
AP	1	NA					
Systemic disease (free)	9	−0.457 (−0.809 to −0.104)	0.011	60.2	0.010	Random	
Systemic disease (mixed)	5	−0.206 (−0.374 to −0.038)	0.016	0.0	0.427	Fixed	
Smoking (free)	7	−0.480 (−0.923 to −0.037)	0.034	69.2	0.003	Random	
Smoking (mixed)	7	−0.227 (−0.387 to −0.068)	0.005	0.0	0.530	Fixed	
L2	9	−0.412 (−0.864–0.040)	0.074	79.7	0.000	Random	0.065
BMI <30	6	−0.541 (−1.213–0.130)	0.114	86.4	0.000	Random	
BMI ≥30	3	−0.201 (−0.562–0.159)	0.273	0.0	0.393	Fixed	
Smoking (free)	7	−0.439 (−1.052–0.174)	0.160	84.4	0.000	Random	
Smoking (Mixed)	2	−0.336 (−0.707–0.034)	0.075	0.0	0.326	Fixed	
A2	9	0.298 (0.117–0.479)	0.001	33.2	0.157	Fixed	0.042
BMI <30	5	0.384 (0.161–0.607)	0.001	54.2	0.068	Fixed	
BMI ≥30	3	0.171 (−0.212–0.554)	0.382	0.0	0.479	Fixed	
SRP	6	0.117 (−0.150–0.384)	0.389	0.0	0.634	Fixed	
SRP + peridontal surgery	2	0.409 (−0.193–1.011)	0.183	74.6	0.047	Random	
SRP + topical antibiotics	1	NA					
Systemic disease (free)	7	0.106 (−0.132–0.344)	0.382	0.0	0.749	Fixed	
Systemic disease (T2DM)	2	0.466 (−0.066–0.997)	0.086	60.9	0.110	Random	

*AP* Aggressive periodontitis, *BMI* body mass index, *CP* chronic periodontitis, *NA* not available, *T2DM* type 2 diabetes mellitus, *SRP* scaling and root planning, *L1* serum levels of leptin in patients with periodontitis versus healthy individuals, *A1* serum levels of adiponectin in patients with periodontitis versus healthy individuals, *L2* serum levels of leptin in patients with periodontitis before versus after periodontal treatment, *A2* serum levels of adiponectin in patients with periodontitis before versus after periodontal treatment

### Meta-regression, Galbraith plots, and sensitivity analyses

Because of the presence of heterogeneities in the subgroup of BMI <30 under all of the four models and the inconsistency of the results between the subgroups of BMI <30 and BMI ≥30 under L1, A1, and A2 models, the meta-regression analyses and Galbraith plots were conducted to investigate the heterogeneity in the subgroup analyses of BMI <30. In addition, the sensitivity analyses were performed to test the stability of the results.

Considering the limitation in the number of studies included in L2 and A2 models (*n* < 10), the meta-regression analyses were performed only in the L1 and A1 models. The results of meta-regression based on the covariates including the type of periodontitis, BMI, systemic conditions, and smoking status failed to find the source of heterogeneity in the subgroup analyses of BMI <30 (*P* > 0.05).

Further, the Galbraith plots showed that the studies performed by Shi et al., Purwar et al., Mendoza-Azpur et al., Thanakun et al., and Karthikeyan et al. (L1) [30, 31, 49, 51, 52]; Mendoza-Azpur et al. and Sete et al. (A1) [16, 31]; and Purwar et al. (L2) [57] were located outside of the two lines in the Galbraith plots of the subgroups of BMI <30. After removing the outliers, the heterogeneities effectively decreased, and the results were not materially changed in the subgroups of BMI <30 (L1: SMD = 0.796, 95% CI: 0.572–1.020, *P* = 0.000; *I* ^2^ = 40.9, *P* = 0.095; A1: SMD = −0.251, 95% CI: −0.408 to −0.095, *P* = 0.002; *I* ^2^ = 13.6, *P* = 0.323; L2: SMD = −0.162, 95% CI: −0.417 to 0.094, *P* = 0.215; *I* ^2^ = 0.0, *P* = 0.556). Additionally, in the L1 model, removing the outlier also eliminated the overall heterogeneity, and the overall result was not materially changed (SMD = −0.175, 95% CI:−0.383 to 0.033, *P* = 0.100; *I* ^2^ = 0.0, *P* = 0.671).

**Fig. 3:**
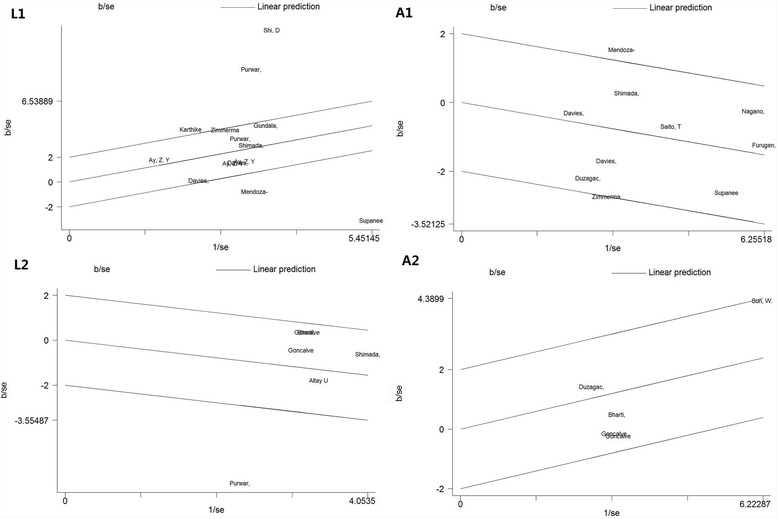
Galbraith plots of the L1, L2, A1 and A2 models in the subgroup of BMI <30. The outlier dots suggest the main contributors to heterogeneity

**Fig. 4:**
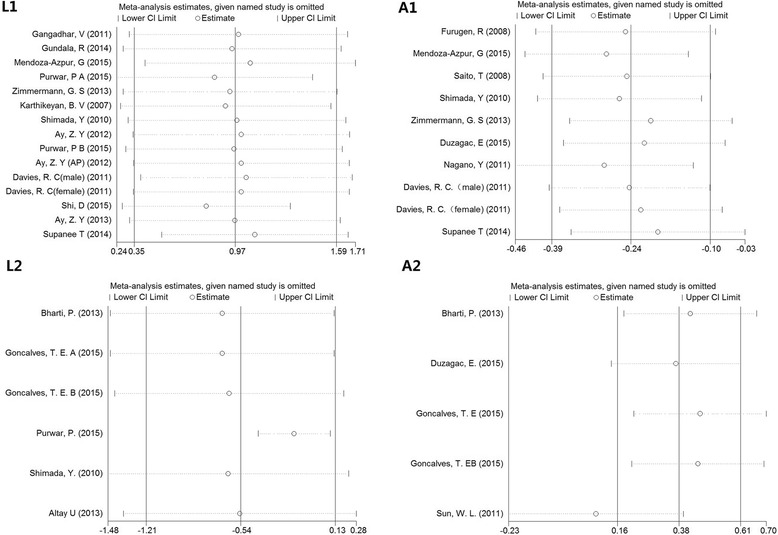
Sensitivity analyses of the L1, L2, A1 and A2 models in the subgroup of BMI <30

The sensitivity analyses were conducted by removing a single study each time and evaluating the influence of each study on the result. The sensitivity analyses proved the robustness of the results of the BMI <30 subgroups under the L1, A1, and L2 models. However, in the A2 model, the sensitivity analysis showed that the study performed by Sun et al. qualitatively changed the pooled SMD [59]. After this study was removed, the results were changed to the insignificant level, and the heterogeneity was eliminated in both overall and BMI <30 analyses (overall: SMD = 0.109, 95% CI: 0.113–0.330, *P* = 0.336; *I* ^2^ = 0.0, *P* = 0.839; BMI <30: SMD = 0.065, 95% CI:−0.238 to 0.369, *P* = 0.673; *I* ^2^ = 0.0, *P* = 0.612). The Galbraith plots and sensitivity analyses are presented in Figs. 3 and 4.

### Publication bias

The Begg’s and Egger’s linear regression tests demonstrated the presence of a publication bias in the A2 model (*P* = 0.042) (Table 2).

## Discussion

To the extent of our knowledge, present study is the first systematic review and meta-analysis quantitatively explored the association between circulating leptin and adiponectin and periodontitis. Our results provided evidence that circulating leptin is elevated and adiponectin is decreased in subjects with BMI < 30 and periodontitis.

Leptin is the main adipokine that is positively related to obesity [60]. The structures of leptin and leptin receptors are similar to the long-chain helical cytokine family, including IL-6, IL-11, and so on. Therefore, leptin is considered to be a functional proinflammatory cytokine, which contributes to both innate and acquired immune response [61]. Previous studies have reported an increased leptin circulation during inflammation periods, which was modulated by lipopolysaccharide (LPS) and cytokines such as TNF and IL-1 [62, 63]. Sachot et al. first revealed that the LPS-induced increase in circulating leptin does not occur in the IL-1β-deficient mice, indicating leptin might exert an inflammatory effect through an IL-1β-dependent mechanism [64]. The change in serum levels of leptin was suggested to modulate the function of immunocytes as well as periodontal cells. In human monocytes, leptin enhanced the *Prevotella intermedia* LPS–induced expression of TNF-α and production in a dose-dependent manner [65]. In periodontal ligament cells, leptin induced the downregulation of TGF-β1, vascular endothelial growth factor, and Runt-related transcription factor-2, indicating an impaired regenerative capacity [66]. Leptin stimulation activated mitogen-activated protein kinase, signal transducer and activator of transcription 1/3, and Akt signaling, and enhanced the expression of MMPs in human gingival fibroblasts [67]. Furthermore, as a pleiotropic adipokine, leptin also exerted an effect on bone metabolism and might contribute to the alveolar bone destruction caused by periodontitis [68]. Through the central nervous system, leptin not only induces bone loss via hypothalamic relay [69] but also inhibits osteogenesis through the sympathetic nervous system [70].

In contrast to leptin, adiponectin, a 244-residue protein produced primarily by adipocytes, was suggested to be inversely associated with obesity [71]. Adiponectin plays an anti-inflammatory role in systemic and local inflammations [72]. It functions by binding to its receptors, adiponectin receptors 1 and 2, which have been found in periodontal tissues [13]. Upon binding to its receptors, adiponectin exerts anti-inflammatory effects such as inhibition of proinflammatory cytokines, induction of anti-inflammatory cytokines, and reduction of adhesion molecule expression, and performs antagonistic effect on toll-like receptors and its ligands [73]. In vivo studies have proved that stimulation with adiponectin improves the regenerative and proliferative capacity of periodontal tissues [24, 74, 75]. Moreover, adiponectin is also involved in the metabolic syndrome and type 2 diabetes mellitus (T2DM). It delays and suppresses the metabolic derangements such as insulin resistance and T2DM because of its remarkable insulin-sensitizing property [76].

Emerging evidence suggests that obesity influences the secretion of adipokines [77–79]. As recommended by the World Health Organization, a BMI ≥30 can be defined as the presence of obesity [31]. In this light, the subgroup analyses were performed based on BMI, which demonstrated a fluctuation in serum levels of leptin and adiponectin in patients with periodontitis having BMI <30 and ≥30. The present study provided evidence that compared with healthy individuals, serum levels of leptin were elevated and those of adiponectin were decreased in patients with periodontitis having BMI <30. Intuitively, this finding was consistent with previous studies, which supported the same tendency of leptin levels during inflammations [80–82]. Nevertheless, the distribution of adiponectin in inflammatory diseases remained controversial. Despite the suppression of secretion of adiponectin in adipocytes by inflammation [73], ample evidence indicates that circulating levels of adiponectin are positively associated with various inflammatory pathologies [83–85]. However, in the present meta-analysis, serum levels of adiponectin were attenuated in patients with periodontitis having BMI <30. Further studies are required to validate this finding.

The sensitivity analyses proved the robustness of the results of BMI <30 subgroup under L1, L2, and A1 models, except for the A2 model. As the sensitivity analysis presented, the study performed by Sun et al. qualitatively influenced the results in the A2 model. This might be caused by the presence of T2DM and the large group size of the study [59]. Adiponectin is an insulin-sensitizing hormone that decreases in metabolic disorders including T2DM [86, 87]. This study hypothesized that the periodontal treatment on patients with T2DM might have improved both the periodontitis and T2DM conditions, thereby resulting in a significantly elevated serum level of adiponectin [88, 89]. Besides that, in the A2 model, the subgroup analysis of the studies involving systemic healthy subjects showed an insignificant result, with no heterogeneity. Moreover, the studies included in the L2 model all enrolled systemic healthy subjects. In this light, it was concluded that serum levels of leptin and adiponectin did not change after periodontal treatment in systemically healthy patients with periodontitis.

Considering the studies evaluating the association of serum levels of cytokines between experimental and control groups as cross-sectional designs, the present study applied the AHRQ form for assessing the quality of the included studies in L1 and A1 models [90]. However, multiple previous studies concerning this issue employed the Newcastle–Ottawa scale (NOS) for the quality assessment [91–93]. The NOS is designed for the meta-analyses of case–control studies and cohort studies, therefore, it is not appropriated for cross-sectional studies.

The present study results, however, has some limitations. First, the quantity of included studies was considered small, especially for the studies enrolling subjects with BMI ≥30. Second, several relevant studies could not be included in present meta-analysis owing to lacking of raw data [94–96], or improper publication formats (Abstract) [97]. Additionally, all the included studies in L1 and A1 models were cross-sectional, and because of the limited number of comparative studies, we treated all the included studies as single-arm designs in L2 and A2 models. Therefore, the cause–effect relationship between periodontitis and serum levels of leptin and adiponectin was rarely presented, and the effect of periodontal treatment on serum levels of leptin and adiponectin was not perfectly elucidated. Further, the statistical heterogeneity observed in the present meta-analysis might impact the veracity of the conclusion. Especially, under the L1 model, significant heterogeneity could be observed in all the subgroups. Although Galbraith plots were conducted to clarify the heterogeneity, the proportion of the outliers (5 out of 14) was considered to be high. Therefore, these results should be carefully interpreted. Moreover, an evident publication bias was observed under the A2 model, which might have distorted the present results because of the presence of the gray (unpublished) manuscripts. Finally, other limitations such as the ethodological diversity in estimating periodontal disease and cytokine levels, the adjustment of the data in the included studies [39–41], the diversity in sample size and the difference in the qualities and methods of the included studies should be noticed when interpreting the results.

## Conclusions

In conclusion, within the limitations of this study, the present meta-analysis supported elevated serum levels of leptin and decreased serum levels of adiponectin in patients with periodontitis compared with controls in the BMI <30 population. In systemically healthy patients with periodontitis, serum levels of leptin and adiponectin did not significantly change after periodontal treatment. Our findings suggested that leptin and adiponectin play as the potential biomarkers for periodontitis. To monitor the adipokines profile may allow clinicians to predict the susceptibility to periodontitis. Further studies are required to explore the mechanism and to clarify the cause–effect relationship between circulating adipokines and periodontitis.

## Abbreviations

AHRQ: Agency for Healthcare Research and Quality
AP: Aggressive periodontitis
BMI: Body mass index
CI: Confidence interval
CP: Chronic periodontitis
Ctr: Control
IL-1: Interleukin-1
LPS: Lipopolysaccharide
M: mean
MINORS: Methodological Index for Nonrandomized Studies
MMP-8: Metalloproteinase-8
NA: Not available
NOS: Newcastle–Ottawa scale
P: Periodontitis
SD: Standard deviation
SMD: Standard mean difference
SRP: Scaling and root planning
T2DM: Type 2 diabetes mellitus
TNF-α: Tumor necrosis factor-α
